# Evaluation of Haptoglobin Genotype and Some Risk Factors of Cancer in Patients with Early Stage Esophageal Cancer

**DOI:** 10.31557/APJCP.2019.20.10.2897

**Published:** 2019

**Authors:** Sara Hosseinzadeh, Reza Alipanah-Moghadam, Fazel Isapanah Amlashi, Ali Nemati

**Affiliations:** 1 *Department of Clinical Biochemistry, School of Medicine, Ardabil University of Medical Sciences, Ardabil, *; 2 *Student Research Committee, Golestan University of Medical Sciences, Gorgan, Iran. *

**Keywords:** Esophageal cancer, haptoglobin ‌genotype, cancer risk factors

## Abstract

**Introduction:**

Esophageal cancer is one of the most lethal gastrointestinal cancers that has a complex and diverse etiology, with several genetic and nutritional factors involved in its etiology. The purpose of this study was to investigate the type of haptoglobin genotype and its relationship with some nutritional and biochemical risk factors affecting the prevalence of esophageal cancer in patients with early stage esophageal cancer.

**Materials and methods:**

In this study, 44 patients (20 males and 24 females) with early stage esophageal cancer and 44 healthy subjects, classified as control group, (19 males and 25 females) were selected. Haptoglobin (HP) genotype was determined employing PCR technique. Nutritional data were analyzed using standard food frequency questionnaire (FFQ) method. Serum levels of malondialdehyde (MDA), nitrate and nitrite were measured employing the colorimetric method. Serum levels of p53 protein were measured using the enzyme-linked immunosorbent assay (ELISA) technique.

**Results:**

The results of our study showed for the first time that HP1-1 genotype was the most prevalent genotype in esophageal cancer patients in Golestan province, Iran. HP2-2 genotype was the most frequent in the control group. Serum levels of MDA were significantly higher in the patients’ group compared to the control group (P˂0.001). Weight and body mass index (BMI) were significantly lower in the patients’ group than the control group (P<0.01). Food frequency analysis revealed that the consumption of fruits and vegetables in the patients’ group was lower than that of the control group (P<0.05).

**Conclusion:**

The results of our study showed for the first time that HP1-1 genotype is the dominant genotype in patients with esophageal cancer in Golestan province. As well, modification of nutritional pattern and consumption of high level of antioxidant compounds can be effective in reducing the prevalence of esophageal cancer in this region.

## Introduction

Esophageal cancer is the eighth most prevalent cancer and the sixth leading cause of death globally. Esophageal cancer is considered as a severe malignancy in view of its prognosis and high mortality rates (Ai et al., 2017; Sohda and Kuwano, 2017). The prevalence of esophageal cancer in Iran is significantly higher compared to other parts of the world. After cancer of the stomach, it is the most common cancer in the Iranian population. Golestan province, one of the northern provinces of Iran, is considered as one of the high-risk areas for esophageal cancer, with an esophageal cancer incidence rate of 100 people per 100,000 people (Gholipour et al., 2016). Esophageal cancer has a complex and diverse etiology, and many genetic and nutritional factors are involved in its etiology. One of the genetic factors that has been considered recently vis-à-vis the etiology of different cancers is the type of haptoglobin genotype (Carter and Worwood, 2007). Haptoglobin is one of the acute phase proteins that are produced primarily by the liver. The most significant role of haptoglobin is to connect to free hemoglobin and prevent its renal excretion, and help regulate the body’s iron cycle. In addition, haptoglobin has several biological properties, including anti-inflammatory properties, angiogenic properties and an inhibitory effect on the cellular and humoral immune systems. The haptoglobin gene has two specific alleles: HP-1 and HP-2; these two alleles produce three genotypes: HP1-1, HP1-2 and HP2-2. Different haptoglobin genotypes produce different proteins with different biological properties (Cigliano et al., 2003; Awadallah and Atoum, 2004; Wobeto et al., 2008). Studies have shown that there is a significant relationship between haptoglobin polymorphism and prevalence of some cancers (Awadallah and Atoum, 2004; Carter and Worwood, 2007).

On the other hand, the most important nutritional factors influencing the incidence of esophageal cancer include alcohol and tobacco consumption, as well as inappropriate food habits such as high salt intake and consumption of hot food and drinks (Rugge et al., 2015; McCormack et al., 2017). Studies have shown that nutritional factors are closely related to oxidative stress as one of the most important factors in the incidence of cancers (Saha et al., 2017). The purpose of this study was to investigate the type of haptoglobin genotype and its relation with some nutritional and biochemical risk factors influencing the prevalence of esophageal cancer in patients with early stage esophageal cancer.

## Materials and Methods

This case control study was conducted on 44 patients (20 males and 24 females) with early stage esophageal cancer and 44 healthy subjects (19 males and 25 females) as control group. A group of patients was selected among people with esophageal cancer who were referred to Sayad Shirazi Hospital and Dezyani Clinic in Gorgan for one year. The physician selected the patients’ group based on the results of endoscopy and paraclinical tests. Patients with early stage esophageal cancer diagnosed for the first time were selected. Patients were enrolled in the study after obtaining written informed consent and before commencement of any treatment and surgery. The inclusion criteria in this study were no history of diabetes, cardiovascular disease, liver and kidney diseases. The control group was selected from healthy people who had no history of malignancy and according to the inclusion criteria. Anthropometric factors (height, weight and BMI) were measured for all patients. Height and weight recorded without shoes and in a light summer uniform using a portable digital scale and a portable digital stadiometer standard technique. Height and weight were measured to the nearest 0.5 cm and 0.1 kg, respectively. BMI was calculated from the patient’s weight and height (kg/m^2^). Food frequency questionnaire (FFQ) was used for evaluating food behavior of patients. Demographic information (age, level of education, sex, job levels, family history of cancer, history of drug use) were taken from the patients with a questionnaire. Educational levels were divided into four groups of illiterates, elementary, secondary, and university, as well as individual job levels were grouped into five groups of office staff, workers, farmers, housewives, and others. Using the “yes and no” questions, the history of cancer in the family and the history of drug use were obtained from the studied groups. After filling out the form containing demographic characteristics and FFQ, blood samples were taken from the subjects in a fasting state. Serum samples were frozen immediately after isolation at -80°C. Serum levels of p53 were measured employing the ELISA method (CUSABIOCSB-E08334h-China). Serum levels of MDA were measured using the colorimetric technique. In this method, MDA was combined with thiobarbituric acid, and the absorbance of the compound was measured at a 530 nm wavelength. Serum levels of nitrate and nitrite were measured employing a standard kit (Cayman Chemical-780001-USA) and colorimetric method.

**Table 1:** The PCR Steps for Amplification of HP1 and HP2 Alleles

Cycle step	Temperature	Time	Cycles
Initial Denaturation	95 °C	5 minutes	1
Denaturation	94 °C	45 seconds	
Annealing	62 °C	1 minute	35
Extension	72 °C	1 minute	
Final Extension	72 °C	7 minutes	1

**Table 2:** Comparison the Mean of Food Frequency Per Week in the Two Study Groups

Food groups (serving)	Patient group N= 44	Control group N= 44	P value	Food groups (serving)	Patient group N= 44	Control group N= 44	P value
Bread and rice	17.73 ± 6.28	13.2 ± 7.04	0.02*	Hot tea	12.88 ± 11.18	8.57 ± 8.38	0.04*
Vegetables	1.59 ± 2.73	3.45 ± 2.23	0.01*	Garlic and onion	4.36 ± 4.36	4.67 ± 3.23	0.7
Fruits	4.16 ± 3.72	8.02 ± 6.53	0.001*	Fish	0.69 ± 1.6	1.01 ± 1.13	0.3
Sausage	0.62 ± 0.43	0.26 ± 0.25	0.01*	Soft drinks	2.69 ± 3.26	1.08 ± 1.76	0.006*
Yogurt	6.34 ± 3.6	7.68 ± 4.1	0.1	Honey	0.52 ± 1.22	1.99 ± 2.17	0.001*
Saturated oil	5.77 ± 4.56	2.24 ± 3.07	0.001*	Milk and cheese	6.12 ± 4.07	7.22 ± 4.13	0.21
Unsaturated oil	3.9 ± 5.22	7.55 ± 3.68	0.001*	Sweetmeat	2.26 ± 3.07	2.63 ± 4.39	0.6

*, significant differences based on independent-samples t-test

Haptoglobin genotype was determined employing PCR technique (Koch et al., 2002). First, the DNA was isolated from the whole blood using a DNA extraction kit (CinnaPure-PR881613-IRAN). A/B and C/D primers were used to determine the haptoglobin genotype. The pair of A/B primers showed the presence of HP-1 alleles by replicating fragment of 1757-bp. The HP-2 allele was characterized by replication of the 3481-bp and 349-bp fragments by the C/D primers. The primer sequences for HP1 and HP2 alleles were A (5’-GAGGGGAGCTTGCCTTTCCATTG-3’), B (5’-GAGATTTTTGAGCCCTGGCTGGT-3’), C (5’-CCTGCCTCGTATTAACTGCACCAT-3’) and D (5’-CCGAGTGCTCCACATAGCCATGT-3’), respectively. The PCR steps are shown in Table 1.

*Statistical analysis*

A t-test was used to determine the mean and examine the relationship between the two groups. Chi-squared test was employed to determine the number and percentage of genotypes in the studied groups and their relationship between the control and patient groups. The Pearson correlation coefficient was used to investigate the relationship between the studied groups. ANOVA test was used to analyze food frequency data. The significance level was considered as p≤0.05.

## Results

The mean age of patients and the control group were 64.73±12.87 and 63.00±12.01, respectively. The average height and weight of the patient and control groups were (159.7±9.93 vs 163.41±8.16) and (54.67±10.6 vs 68.36±7.53), respectively. The mean values of BMI in the patients and the control groups were 21.56±4.29 and 24.6±5.18 (kg/m2), respectively, (P<0.01). The results of our study showed that HP1-1 genotype was the most abundant genotype in the patients’ group with twenty-four subjects (57.1%), and the lowest in the control group with nine subjects (21.4%). Furthermore, HP1-2 genotype had the lowest frequency in the patients’ group with 6 patients (14.3%); the rate of this genotype in the control group was nine (21.4%). In addition, HP2-2 genotype was the most frequent in the control group with 24 people (57.1%); however, this genotype was found in 12 people (28.6%) in the patients’ group. Based on our study, weight and BMI were significantly lower in the patients’ group than the control group (P˂0.01). Based on the chi-squared test, our results revealed that there was a significant relationship among all genotypes in the patients and control groups (P˂0.01). Our results also showed that serum levels of MDA in the patients’ group were significantly higher compared to the control group (6.42±1.26 vs 4.08±0.7 nmol/ml) (P˂0.001). Based on t-test, although serum levels of p53 protein were lower in the patients’ group than the control group (38.31±30.9 vs 40.13±47.63 pg/ml), however, we did not observe any significant correlation between serum levels of p53 protein in the two groups. Based on the Pearson statistical test, there was a reverse relationship between serum levels of MDA and serum levels of p53 in the patients’ group; this relationship was not significant. According to the results of our study, there was a significant relationship between serum levels of nitrate in the patients’ group and control group. The serum levels of nitrate in the control group were significantly higher than that of the patients’ group (11.15±9.43 vs 4.51±5.19 µmol) (P<0.001). Our results showed that serum levels of nitrite in the control group were significantly higher than that in the patients’ group (25.15±9.14 vs 18.8±12.68 µmol) and there was a significant relationship between the two groups in this case (P<0.01). Also, our study revealed that there was a reverse relationship between serum levels of p53 and serum levels of nitrate and nitrite in the patients’ group, which was not statistically significant. Our results also revealed that there was a reverse relationship between BMI and serum levels of nitrite, p53 and MDA in the patients’ group. The results of the demographic factor in the studied groups showed that a significant relationship between education levels, job levels, and family history in both groups (p<0.05). Our results showed that the changes in quantitative indices between the two groups were almost the same based on the Pearson’s statistical test. The results of statistical analysis of food frequency showed that the consumption of fruit and vegetables in the patients’ group was lower than that of the control group (Table 2) (P<0.05). 

## Discussion

Esophageal Cancer, which is one of the deadliest forms of cancer in the world, causes the death of many people annually (Wong et al., 2018). Interactions between genetic and environmental factors have been shown to play a potential role in the development and progression of esophageal cancer. It seems that identification of the effective genetic predispositions for esophageal cancer and control of the environmental factors that affect this disease can somewhat help in the successful prevention and control of this disorder (Dong et al., 2018). One of the genetic factors that has attracted much attention in recent years vis-à-vis various types of cancer is the type of haptoglobin genotype. It has been shown that haptoglobin genotype is related to the prevalence and progression of various types of cancer (Carter and Worwood, 2007). The results of our study also indicated that there was a significant correlation between haptoglobin genotype and esophageal cancer and that HP1-1 genotype was more prevalent than HP1-2 and HP2-2 genotypes in patients with esophageal cancer. In consonance with our study, several studies have shown that the specific genotype of haptoglobin has a higher prevalence in some cancers (Lai et al., 2010; Levy et al., 2010). In the study of Mahmud et al., (2007), on uterine cancer, the HP1-1 genotype was more prevalent than other genotypes. Also, in the study of Lee et al., (2009), the HP2-2 genotype of haptoglobin was the most frequent genotype in tracheal cancer, and this genotype was also associated with the severity of tracheal cancer. In contrast, in some studies, there was no significant relationship between haptoglobin genotype and cancer incidence (Mavondo et al., 2012). It seems that in addition to the prevalence of specific haptoglobin genotype in some cancers regarding the geographical distribution of haptoglobin alleles in different parts of the world, the characteristics of the haptoglobin genotype is also efficacious. It has been demonstrated that the innate immune response to tumor growth in individuals with the HP-1 allele is reduced compared to those with the HP-2 allele, which could be the reason for tumor growth (Abdullah et al., 2009; Bicho et al., 2013). In our study, the relationship between esophageal cancer and a specific type of haptoglobin genotype could be very important because this relationship reflects the inherent potential of these individuals to develop esophageal cancer. Consequently, it is necessary to minimize the effects of genetic factors by identifying efficacious environmental factors associated with the occurrence of esophageal cancer. Oxidative stress induced by environmental factors is one of the primary factors that could be very effective in the development of most cancers (Alshiek et al., 2017; Andrisic et al., 2018). It has been demonstrated that serum levels of MDA, as an indicator of oxidative stress, are much higher than normal in most cancers (Didziapetriene et al., 2014; Majidi et al., 2017). Our results revealed that serum levels of MDA in the patients’ group were significantly higher than that of the control group, indicating the presence of oxidative stress in the patients’ group. It has been shown that oxidative stress provides the basis for the onset and progression of cancer by making structural changes in DNA, as well as activates some oncogenes (Gorrini et al., 2013). Moreover, MDA, as an end product of lipid peroxidation, has potentially toxic effects and can induce genetic mutations in the DNA structure by binding to DNA (Del Rio et al., 2005). Therefore, since oxidative stress is one of the most significant factors contributing to the pathogenesis of various cancers, the control of oxidative stress can be very efficacious in preventing esophageal cancer (Andrisic et al., 2018). One of the effective environmental factors associated with oxidative stress and prevalence of various cancers is inappropriate nutritional behaviors. It has been demonstrated that inappropriate nutritional behaviors such as decrease in the use of antioxidants in a variety of ways including increase in oxidative stress can provide a very favorable environment for the development of various types of cancer (Saha et al., 2017; Seiler et al., 2018). The study of nutritional behaviors in both groups (patients and control) in our study showed that fruit and vegetable consumption in the patients’ group was significantly lower compared to the control group. Studies have shown that one of the most important factors contributing to the increase in oxidative stress is reduction in the consumption of antioxidant compounds and there is a clear relationship between constant consumption of antioxidant compounds such as fruit and vegetables and reduction of oxidative stress (Khan et al., 2014; Kaluza et al., 2017). Similarly, reduction in the consumption of these compounds could be associated with the development of various cancers, especially digestive cancers (Turati et al., 2015). This has also been confirmed in our study. Based on the results of our study, the consumption of salt, hot tea, soft drinks, Deli meats and saturated oil in the patients’ group was significantly higher than that of the control group. Some studies have demonstrated that inappropriate nutritional behaviors such as high salt intake and hot drinks can be associated with the incidence of gastrointestinal cancers (Shivanna and Urooj, 2016; Xie et al., 2016). It has been shown that the consumption of highly concentrated salt can induce mucosal membrane damage, stimulate inflammatory responses and increase the proliferation of epithelial cells, and subsequently increase the probability of carcinogenic genetic mutations (Wang et al., 2009). It has also been demonstrated that the consumption of hot beverages increases the risk of esophageal cancer by 2-4 times (Castellsague et al., 2000). Kamangar and Freedman (2018) showed that esophageal cancer is closely related to hot drinks. Hot drinks with precancerous lesions can be involved in the development of esophageal cancers (Castellsague et al., 2000). Consequently, according to our study, which indicated that there was a close correlation between inappropriate nutritional behaviors and esophageal cancer, one of the essential elements for prevention of esophageal cancer is to avoid inappropriate nutritional behaviors. One of the interesting results of our study was the lower serum levels of nitrate and nitrite in the patients’ group compared to the control group. Reduction in serum levels of nitrate and nitrite in this study could be due to decrease in consumption of fruit and vegetables (as the main sources of nitrate and nitrite) or increased activity of the nitric oxidase enzyme in these patients. It has been shown that in some cancers, the activity of this enzyme increases and it converts nitrite to nitric oxide (Badawi et al., 1998; Hord et al., 2009).

In conclusion, overall, the results of our study showed for the first time that HP1-1 genotype is the dominant genotype in patients with esophageal cancer in Golestan province (as the main area of esophageal cancer in Iran), and this genotype is closely associated with esophageal cancer. Additionally, oxidative stress level in these patients was much higher than that of the control group, which could be due to the low consumption of antioxidant compounds. Therefore, taking into cognizance the type of haptoglobin genotype of patients in this region, modification of nutritional pattern and consumption of high level of antioxidant compounds as well as reduction of oxidative stress can be effective in reducing the prevalence of esophageal cancer in this region.
